# Evaluating the toxicological effects of DEHP exposure on inflammatory bowel disease: insights from comprehensive network analysis

**DOI:** 10.1097/JS9.0000000000002780

**Published:** 2025-07-17

**Authors:** Gaoquan Cao, Shuai Liu, Yihui Qian, Jingyi Gan, Hui Liang, Shuman Guo, Xiuqin Zhang, Ziqian Liu, Xinrui Liu, Jie Gao, Linfeng Jin, Xiajing Yu, Kaili Liao, Xiaozhong Wang

**Affiliations:** a Jiangxi Province Key Laboratory of Immunology and Inflammation, Jiangxi Provincial Clinical Research Center for Laboratory Medicine, Department of Clinical Laboratory, The Second Affiliated Hospital, Jiangxi Medical College, Nanchang University, Nanchang, Jiangxi, China; b The Fourth School of Clinical Medicine, Jiangxi Medical College, Nanchang University, Xuefu Road,Nanchang, Jiangxi, China; c The Second Affiliated Hospital, Jiangxi Medical College, Nanchang University, Nanchang, Jiangxi, China; d The First Affiliated Hospital, Jiangxi Medical College, Nanchang University, Xuefu Road, Nanchang, Jiangxi, China; e Queen Mary college, Jiangxi Medical College, Nanchang University, Xuefu Road, Nanchang, Jiangxi, China; f School of Public Health, Jiangxi Medical College, Nanchang University, Xuefu Road, Nanchang, Jiangxi, China

## Abstract

**Figure:**
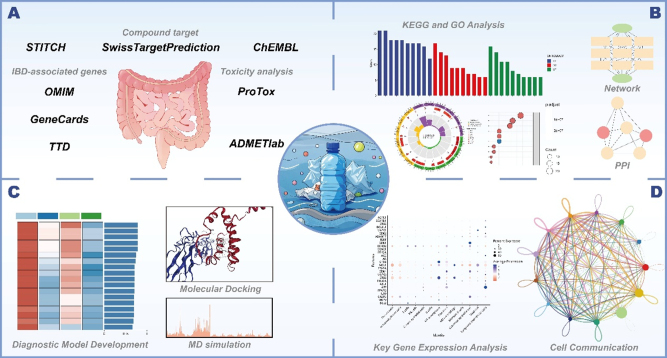

(A) Integration of multiple databases to identify DEHP-associated IBD targets; (B) Utilization of KEGG/GO enrichment analysis and PPI network analysis to determine functional enrichment patterns and key targets in DEHP-associated IBD; (C) Application of machine learning, molecular docking and MD simulations to assess diagnostic value of targets and characterize docking complexes; (D) Single-cell transcriptomic profiling to explore expression signatures of DEHP-associated IBD targets.

Inflammatory bowel disease (IBD), a chronic immune-mediated digestive system disorder, primarily comprises Crohn’s disease and ulcerative colitis. This disease can be diagnosed across all age groups, with environmental exposure recognized as one of its critical etiological factors^[1]^. Di(2-ethylhexyl) phthalate (DEHP), one of the most widely used plasticizers in polyvinyl chloride production, significantly enhances the flexibility and practicality of products. However, as an endocrine-disrupting chemical, DEHP readily migrates from plastic matrices into the environment. Its exposure disrupts gut microbiota homeostasis, promotes metabolic dysregulation, and exacerbates intestinal inflammation^[2-4]^. Therefore, this study employs network toxicology analysis integrating toxicology and bioinformatics to explore the potential molecular mechanisms of DEHP in inflammatory bowel disease. In accordance with the TITAN Guideline, we declare that no artificial intelligence was used in this study^[5]^.

This study systematically evaluated the toxic mechanisms and diagnostic potential of DEHP in inflammatory bowel disease IBD by integrating computational toxicology, bioinformatics, and machine learning approaches. First, the toxicological characteristics of DEHP were analyzed based on the PubChem, ADMETlab-3, and ProTox-III platforms, with its gastrointestinal toxicity validated through literature screening in PubMed and Web of Science. Subsequently, 116 DEHP target genes were identified via ChEMBL, STITCH, and SwissTargetPrediction, and intersecting with 4240 IBD-related genes from GeneCards, OMIM, and TTD databases yielded 89 key target genes. KEGG/GO enrichment analyses were performed using clusterProfiler to reveal biological functions, while a protein-protein interaction (PPI) network was constructed via the STRING database (confidence ≥0.4), with core targets (e.g., BCL2, MMP9) identified using the CytoHubba plugin in Cytoscape 3.9.1. Molecular docking (CB-Dock2 platform, binding energy <-5 kcal/mol) and iMODS molecular dynamics simulation were used to validate target binding stability. Based on the GSE75214 dataset, 107 ensemble models incorporating 12 machine learning algorithms were constructed, with the optimal diagnostic model selected after validation in three independent cohorts. Finally, single-cell RNA sequencing data from GSE214695 were used to dissect cell-specific expression and communication networks of target genes in IBD colonic tissues. This full-process integration of multi-omics data and computational simulations systematically elucidates the toxic mechanisms of DEHP in IBD and establishes a diagnostic model.

**HIGHLIGHTS**

- Identified 89 DEHP-associated IBD targets enriched in apoptosis, glycosylation, and immune pathways via multi-database network analysis.
- BCL2, MMP9, IL10, CASP3, and PPARG emerged as core targets with stable DEHP binding confirmed by molecular docking and dynamics simulations.
- Machine learning diagnostic model (glmBoost + NaiveBayes) achieved high accuracy (AUC = 0.896) for IBD detection using DEHP-related targets.
- Single-cell transcriptomics revealed hub gene expression in immune cells (e.g., M1 macrophages), linking DEHP to IBD pathogenesis.

This study revealed the associative mechanisms between DEHP and IBD through multidimensional analysis. First, the SDF/SMILES structures of DEHP were obtained from PubChem, followed by toxicity assessment using ProTox-III and ADMETlab-3.0 (Fig. 1A; Supplementary Digital Content, available at: Figure S1, http://links.lww.com/JS9/E745, Figure S2, http://links.lww.com/JS9/E746, Figure S3, http://links.lww.com/JS9/E747), which was combined with literature validation to confirm its role as a potential risk factor for IBD. Subsequently, 116 DEHP-associated target genes were screened via ChEMBL, STITCH, and SwissTargetPrediction (Fig. 1B). Meanwhile, IBD-related genes from GeneCards, OMIM, and TTD databases were integrated, and intersection analysis identified 89 key overlapping target genes (Fig. 1C). In the KEGG enrichment analysis, the AGE-RAGE signaling pathway in diabetic complications, p53 signaling pathway, and apoptosis pathway were found to be the most significantly enriched (Fig. 1D,E). In the GO enrichment analysis, regulation of inflammatory response, GABA-A receptor complex, and GABA-gated chloride channel activity were identified as the most prominent terms, further corroborating the results of the KEGG enrichment analysis (Fig. 1F,I).

**Figure 1.**
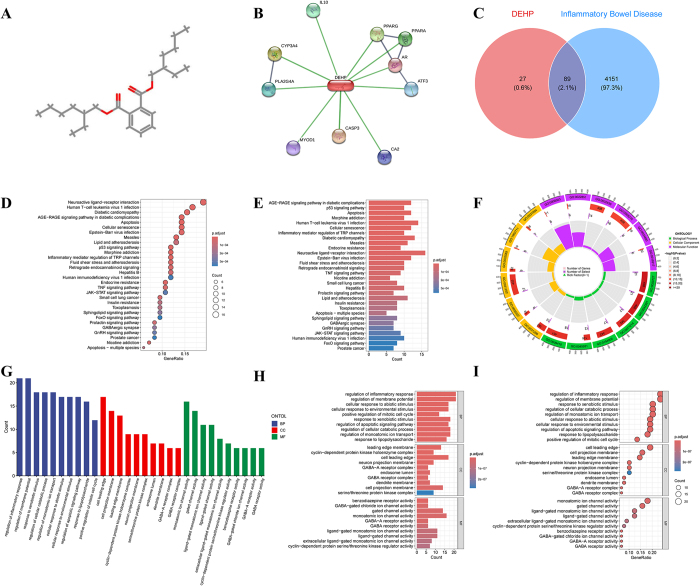
Identification of DEHP-related IBD targets and KEGG/GO enrichment analysis. (A) Molecular structure of DEHP; (B) DEHP targets in the STITCH database; (C) Intersection analysis between DEHP targets and IBD-related genes to identify DEHP-associated IBD targets; (D-E) KEGG enrichment analysis results; (F-I) GO enrichment analysis results.

We input the screened DEHP-related IBD target genes into the STITCH database to construct a protein-protein interaction (PPI) network, and visualized the DEHP-target-IBD network using Cytoscape software (Version 3.9.1). Through topological analysis with the CytoHubba plugin in Cytoscape, target genes were ranked based on degree centrality, identifying five hub genes—BCL2, MMP9, IL10, CASP3, and PPARG—as the most critical nodes in the network (Supplementary Digital Content Figure S4, available at: http://links.lww.com/JS9/E748). To further explore the binding potential of DEHP with these hub targets, systematic molecular docking analyses were performed. Results showed significant binding affinities between DEHP and all targets, with Vina scores of −5.6, −5.2, −6.7, −6.0, and −7.9 kcal/mol for BCL2, MMP9, IL10, CASP3, and PPARG, respectively (Fig. 2, Table 1). Normal mode analysis of top-ranked docking complexes was conducted using the iMODS server, evaluating stability through four key parameters: deformability, eigenvalues, covariance maps, and elastic network models. Backbone deformability analysis revealed low overall deformation in all complexes except the PPARG-DEHP complex, which exhibited moderate deformation (Supplementary Digital Content Figure S5, available at: http://links.lww.com/JS9/E749).

**Figure 2.**
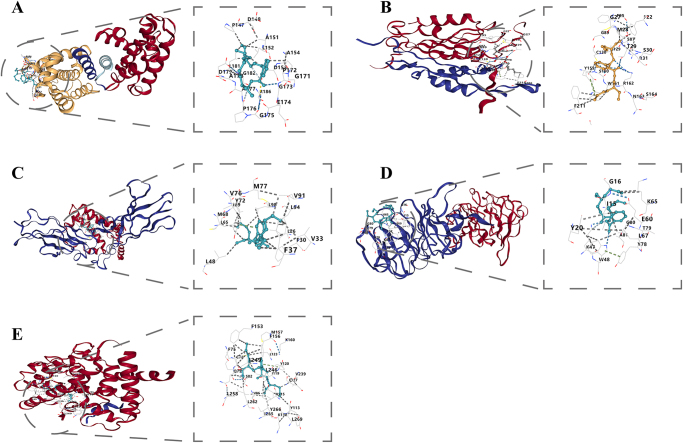
Molecular docking results of DEHP with core DEHP-associated IBD targets. (A) DEHP-BCL2 molecular docking results; (B) DEHP-CAPS3 molecular docking results; (C) DEHP-IL10 molecular docking results; (D) DEHP-MMP9 molecular docking results; (E) DEHP-PPARG molecular docking results.

**Table 1:** Molecular docking results between core DEHP-associated IBD targets and DEHP

	Cavities_volume	center_x	center_y	center_z	score	contact_resi
BCL2	133	−13.974	8.709	9.933	−5.6	PRO:147:B,ASP:148:B,ARG:150:B,ALA:151:B,LEU:152:B,ALA:154:B,ASP:155:B,GLY:171:B,PRO:172:B,GLY:173:B,GLU:174:B,GLY:175:B,PRO:176:B,ALA:177:B,ALA:178:B,ASP:179:B,PRO:180:B,LEU:181:B,GLY:182:B,ARG:186:B,TRP:283:B,ALA:284:B,THR:287:B
CAPS3	139	26.003	95.397	0.432	−5.4	GLU:91:A,GLY:92:A,ILE:93:A,ASP:102:A,LEU:103:A,LYS:104:A,THR:107:A,ASN:108:A,ARG:131:A,GLU:145:B,TYR:150:B,TYR:152:B,PRO:156:B,VAL:221:B,MET:223:B
IL10	461	11.743	−2.475	18.966	−6.7	LEU:23:L,LEU:26:L,ARG:27:L,PHE:30:L,VAL:33:L,PHE:37:L,LEU:48:L,LEU:52:L,PHE:56:L,LEU:65:L,MET:68:L,ILE:69:L,TYR:72:L,VAL:76:L,MET:77:L,ALA:80:L,VAL:91:L,LEU:94:L,LEU:98:L,LEU:101:L,LEU:105:L,ASP:100:R
MMP9	222	−4.05	−58.622	−26.872	−5.2	ILE:15:B,GLY:16:B,ASN:17:B,GLN:18:B,TYR:20:B,ARG:29:B,ASP:46:B,LYS:47:B,TRP:48:B,GLU:60:B,SER:64:B,LYS:65:B,LYS:66:B,LEU:67:B,TYR:78:B,THR:79:B,GLY:80:B,ALA:81:B
PPARG	2940	−22.469	−17.683	9.323	−7.9	ALA:71:A,ILE:74:A,PHE:75:A,GLN:76:A,CYS:78:A,GLN:79:A,ARG:81:A,SER:82:A,ALA:85:A,VAL:86:A,LYS:112:A,TYR:113:A,GLY:114:A,VAL:115:A,GLU:117:A,ILE:118:A,ILE:119:A,TYR:120:A,LEU:123:A,LEU:146:A,LEU:149:A,PHE:153:A,PHE:156:A,MET:157:A,LYS:160:A,VAL:239:A,VAL:243:A,LEU:246:A,ILE:249:A,LEU:258:A,LEU:262:A,ILE:265:A,TYR:266:A,LEU:269:A

Evaluation of 107 combined models using 12 machine learning algorithms revealed that the glmBoost + NaiveBayes combined algorithm exhibited the optimal overall performance in both training and test sets, with an average AUC value of 0.896, ranking first among all models (Fig. 3). Next, in single-cell transcriptome analysis, hub genes were highly expressed in M1 macrophages but lowly expressed in plasma cells (Supplementary Digital Content Figure S6A-D, available at: http://links.lww.com/JS9/E750). Additionally, intercellular communication analysis indicated that T cells, M1 macrophages, and plasma cells dominated the cellular signaling network, suggesting their potential involvement in mediating DEHP-induced IBD risk (Supplementary Digital Content, available at: Figure S6E-F, http://links.lww.com/JS9/E750, Figure S7, http://links.lww.com/JS9/E751, Figure S8, http://links.lww.com/JS9/E752, Figure S9, http://links.lww.com/JS9/E753).

**Figure 3.**
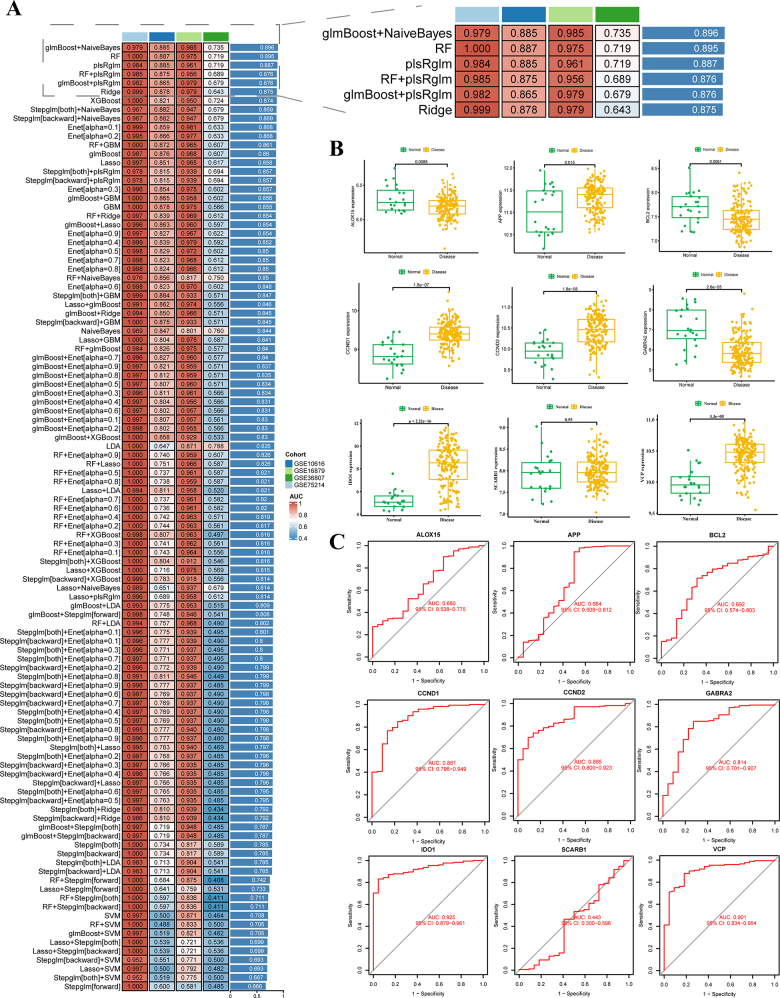
Construction of DEHP-associated IBD diagnostic models based on 107 combined machine learning algorithms. (A) AUC results of different algorithm combinations; (B) Differential expression analysis of modeling genes in the glmBoost + NaiveBayes model; (C) ROC analysis of modeling genes in the glmBoost + NaiveBayes model.

This study systematically elucidated the potential association between DEHP exposure and IBD through an integrated approach combining network toxicology, machine learning, single-cell transcriptomics, and molecular docking, identifying and validating key DEHP-related IBD targets such as BCL2, MMP9, IL10, CASP3, and PPARG. Moreover, these hub targets demonstrated significant potential as diagnostic biomarkers for IBD and provided actionable insights for developing targeted therapeutic strategies. The multidimensional framework established in this study not only deciphered the molecular interactions between environmental toxins and IBD pathogenesis but also advanced the development of precision medicine for IBD.

## Data Availability

All the data and materials are readily available. Please contact us, if it is needed.

## Research registration unique identifying number (UIN)

No humans were involved in this study.
